# Changes of pathological and physiological indicators affecting drug metabolism in rats after acute exposure to high altitude

**DOI:** 10.3892/etm.2014.2049

**Published:** 2014-11-04

**Authors:** WENBIN LI, RONG WANG, HUA XIE, JUANHONG ZHANG, ZHENGPING JIA

**Affiliations:** Department of Pharmacy, Key Laboratory of Plateau Environmental Damage Control, Lanzhou General Hospital of Lanzhou Command, Lanzhou, Gansu 730050, P.R. China

**Keywords:** acute exposure to high altitude, blood gas analysis, biochemical indicators, pathological changes, Wistar rats

## Abstract

High altitude environments cause the human body to undergo a series of pathological, physiological and biochemical changes, which have a certain effect on drug pharmacokinetics. The objective of the present study was to observe changes in factors affecting pharmacokinetics in rats following acute exposure to high altitude and return to low altitude. A total of 21 male Wistar rats were randomly assigned to three groups. The rats in group A were maintained at low altitude in Shanghai, 55 m above sea level; those in group B were acutely exposed to high altitude in Maqu, Gansu, 4,010 m above sea level; and those in group C were acutely exposed to high altitude and then returned to low altitude. Blood was collected from the orbit for the analysis of significant biochemical indicators and from the abdominal aorta for blood gas analysis. Brain, lung and kidney tissues were removed to observe pathological changes. In group B, the pH, buffer base (BB), base excess (BE), total carbon dioxide content (ctCO_2_), oxygen saturation of arterial blood (sO_2_), oxygen tension of arterial blood (pO_2_), serum sodium (Na^+^) concentration, lactate dehydrogenase (LDH) activity and total protein (TP) level were significantly reduced, and the carbon dioxide tension of arterial blood (pCO_2_), serum chloride (Cl^−^) concentration, serum total bilirubin (TBIL) level and alkaline phosphatase (ALP) activity were significantly increased compared with those in group A (P<0.05). In group C, the pH, BB, BE, sO_2_, pO_2_, hemoglobin (Hb) level, serum Na^+^ concentration, LDH activity and TP level were significantly reduced, and the pCO_2_, serum Cl^−^ concentration, alanine transaminase activity, TBIL and urea levels were significantly increased (P<0.05) compared with those in group A. The Hb and ALP levels in group C were significantly lower than those in group B (P<0.05); and the TP, TBIL and urea levels in group C were significantly higher than those in group B (P<0.05). Pathological observation revealed that the alveolar wall was hyperemic, edematous and incrassate, the alveolar epithelium was hyperplastic and infiltrated with neutrophilic granulocytes and the alveolar septum was widened; brain neurons were edematous with enlarged perivascular spaces, and hippocampal neurons were metamorphic and karyopyknotic; and kidney mesangial cells were hyperplastic, both following acute exposure to high altitude and after returning to low altitude. In conclusion, blood gas indices, biochemical indicators and functions of the heart, liver, kidney were significantly changed, and marked pathological changes occurred in the brain, liver and kidney following acute exposure to high altitude and also after returning to low altitude. These changes are likely to seriously affect the pharmacokinetics of drugs.

## Introduction

Hypoxia is a very important factor affecting the health and life activities of individuals at high altitude, which has serious impacts on physiology and induces pathological changes in the body (1). There is a close link between these changes and drug pharmacokinetics, for example, blood pH has an impact on the absorption and distribution of drugs (2); hypoxemia and acid-base imbalance have significant effects on the absorption, distribution, metabolism and excretion of drugs. Therefore, arterial blood gas analysis is of great significance for pharmacokinetics. In addition, protein binding rates influence the distribution of drugs and plasma drug concentrations; cardiac function affects blood rheology and distribution; and liver and renal function influence drug metabolism and excretion (3), which seriously affect the therapeutic effects and side-effects of drugs (2). As a result, studies concerning the physiological and pathological changes in rats from low altitude that are acutely exposed to high altitude lay an important foundation for further research on the impacts of high altitude and low oxygen levels on drug pharmacokinetics. They may provide guidance for the adjustment of drug usage and dosage in individuals who are acutely exposed to high altitude or live at high altitude for a long time, with the aim of achieving optimal treatment outcome and reduced drug side-effects.

In the present study, the physiological and pathological effects in Wistar rats of acute exposure to a high altitude of 4,010 m followed by a return to low altitude were investigated in order to identify the associations between high altitude and drug pharmacokinetics.

## Materials and methods

### Equipment and reagents

An automatic blood gas system (ABL80; Radiometer Medical, Brønshoj, Denmark), automatic biochemistry analyzer (LX20; Beckman Coulter, Inc., Brea, CA, USA), high speed centrifuge (TGL-16B; Shenzhen Anke High-Tech Co., Ltd., Shenzhen, China), hand-held GPS (Planet Neptun 500E, China Magellan Corporation, Beijing) and electron microscope (BX-51; Olympus, Tokyo, Japan) were used.

### Animals

A total of 21 healthy and clean male Wistar rats (Shanghai SLAC Laboratory Animal Co. Ltd., Shanghai, China; Certificate number: 2007000524909) were used in the study. The rats weighed 200±20 g and all originated from a low altitude area. They were randomly divided into group A, which was maintained at low altitude (Shanghai, 31°30′NW, 121°52′EL; 55 m above sea level; 24°C; pressure, 95.6 kPa; relative humidity, 73%); group B, which was acutely exposed to high altitude (Maqu, Gansu, 33°97′NW, 102°04′EL; 4,010 m above sea level; −2°C; pressure, 62.1 kPa; relative humidity, 48%) and group C, which was taken to high altitude and then back to low altitude (n=7 per group). This study was approved by the Ethical Committee of Lanzhou General Hospital of Lanzhou Command (Lanzhou, China).

### Animal treatment methods

The rats in group A were normally fed at low altitude (Shanghai; 55 m) and had an average weight of 203 g. The rats in group B were acutely exposed to high altitude (4,010 m), where they were fed normally for 72 h, and had an average weight of 198 g. The rats in group C were fed normally during acute exposure to high altitude (4,010 m) for 72 h, and then returned to low altitude where they were normally fed for a further 24 h; these rats had an average weight of 207 g. The rats were transported by aviation in a semi-enclosed polypropylene cage. In each group, examination of the rats began after fasting for 12 h.

### Blood gas analysis

In this study, 1 ml blood was taken from the abdominal aorta following anesthetization by the injection of 1 ml 10% chloral hydrate into the peritoneal cavity. Blood gas analysis was carried out immediately using the automatic blood gas system. The indicators measured were pH, buffer base (BB), base excess (BE), content of total carbon dioxide (ctCO_2_), oxygen saturation of arterial blood (sO_2_), carbon dioxide tension of arterial blood (pCO2), oxygen tension of arterial blood (pO_2_), hemoglobin (Hb) level, sodium ion concentration (cNa+), potassium ion concentration (cK^+^) and chloride ion concentration (cCl^−^).

### Biochemical indicator analysis

In this study, 3 ml blood was collected from the superior vena orbitalis posterior into centrifuge tubes that contained heparin sodium. The samples were centrifuged at 664 × g for 10 min at room temperature and then analyzed with the automatic biochemistry analyzer (4,5). The biochemical indicators were lactate dehydrogenase (LHD), alanine aminotransferase (ALT), aspartate aminotransferase (AST), alkaline phosphatase (ALP), total protein (TP), total bilirubin (TBIL), glucose (GLU), urea and uric acid (UA) (4,6,7).

### Pathological changes

Cerebrum, lungs and kidneys were collected and put into phosphate-buffered solution containing 10% formaldehyde solution for longer than 24 h. The samples were fixed thoroughly. Both kidneys were cut in half horizontally from the midline from the lateral border to the renal hilum (deep into the kidney calices) and were then stained with hematoxylin and eosin. Pathological changes were observed under the electron microscope.

### Data analysis

All data are presented as the mean ± standard deviation. Analysis of statistical significance was performed, and P<0.05 was considered to indicate a statistically significant result. An analysis of variance (SNK-q test) was used to perform multiple comparison between the three groups. The analysis was carried out using SPSS software, version 13.0 (SPSS, Inc., Chicago, IL, USA).

## Results

### Comparison of blood gas analysis

The results of the blood gas analysis are presented in Table I. Compared with the values in group A, the pH, BB, BE, ctCO_2_, sO_2_, pO_2_ and cNa^+^ values of group B were significantly decreased by 2.43, 630, 311, 11.48, 91.38, 76.22 and 2.82% respectively, while the pCO_2_ and cCl^−^ values significantly increased by 47.40 and 6.76%, respectively. In group C, the pH, BB, BE, sO_2_, pO_2_, Hb and cNa^+^ values were significantly decreased by 3.24, 542.00, 296.00, 92.89, 89.46, 32.32 and 4.20%, respectively, while the pCO_2_ and cCl^−^ values were significantly increased by 75.49 and 4.25%, respectively. The Hb level of group C was decreased by 25.82% compared with that in group B.

Statistical analysis demonstrated that the pH, BB, BE, sO_2_ and pO_2_ were significantly reduced while ctCO_2_ was significantly increased following the acute exposure of the rats to high altitude.

### Comparison of main biochemical indicators

Biochemical indicators are main indicators for evaluating the function of major organs. The biochemical indicator results are presented in Table II. Analysis of the results shows that the LHD and TP levels of group B were significantly decreased by 58.44 and 26.82%, respectively, compared with those in group A, while the TBIL and ALP levels were severely increased by 338.00 and 24.94%, respectively. The LHD and TP levels of group C were significantly decreased by 5.98 and 17.41%, respectively, compared with those in group A, while the TBIL and urea levels increased by 478 and 36.20%, respectively. The ALP level of group C decreased by 19.19% compared with that in group B, whereas the TP, TBIL and urea levels significantly increased by 12.85, 31.93 and 40.32%, respectively.

### Comparison of pathological changes

#### Results of hematoxylin and eosin staining in lung alveoli

**Figure 1:**
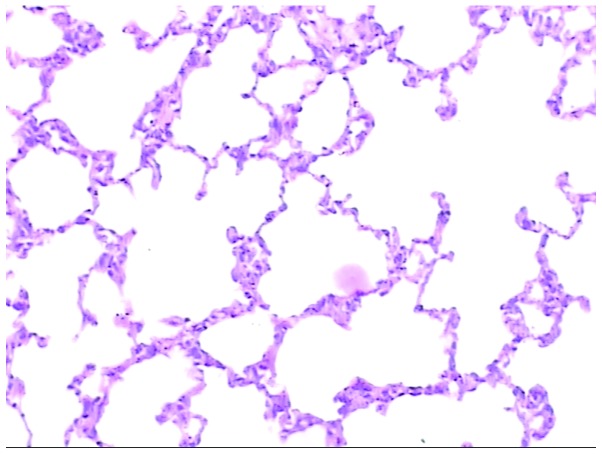
Hematoxylin and eosin staining showing alveoli of a rat at low altitude (magnification, ×100).

**Figure 2:**
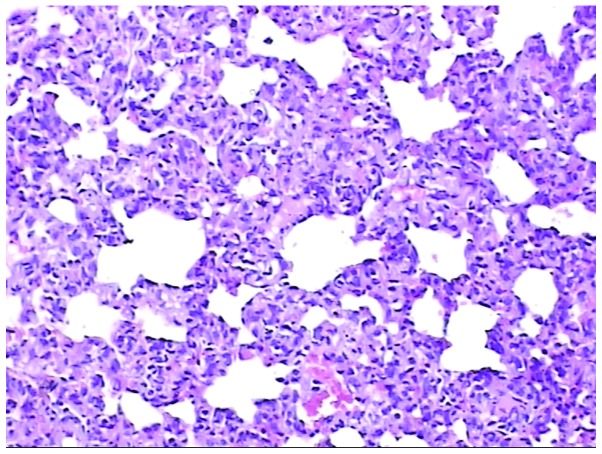
Hematoxylin and eosin staining showing alveoli of a rat at high altitude (magnification, ×100).

**Figure 3:**
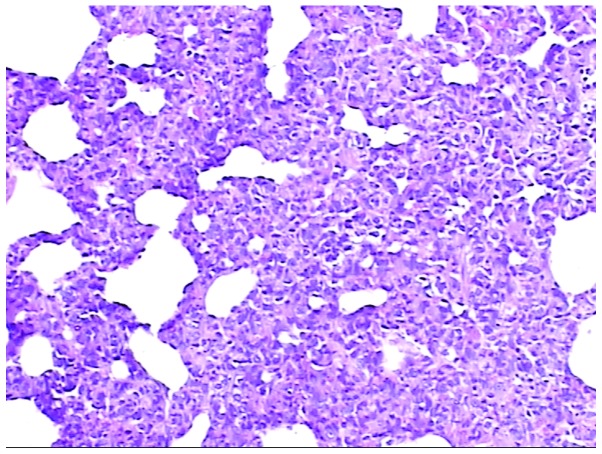
Hematoxylin and eosin staining showing alveoli of a rat that returned to low altitude from high altitude (magnification, ×100).

The results show significant pathological changes in the alveoli among the groups. Fig. 1 shows normal alveolar tissue, whereas Fig. 2 shows pathological alveolar tissue observed following acute exposure to high altitude. Following acute exposure to high altitude, the alveolar wall became hyperemic, edematous and incrassate; the alveolar epithelium became hyperplastic and neutrophilic granulocyte infiltrates were present. Fig. 3 shows pathological alveolar tissue from a rat that had returned to low altitude from high altitude. The alveolar wall was hyperemic, edematous and incrassate; the alveolar epithelium was hyperplastic, and the alveolar septum was widened.

#### Results of hematoxylin and eosin staining of brain tissue

**Figure 4:**
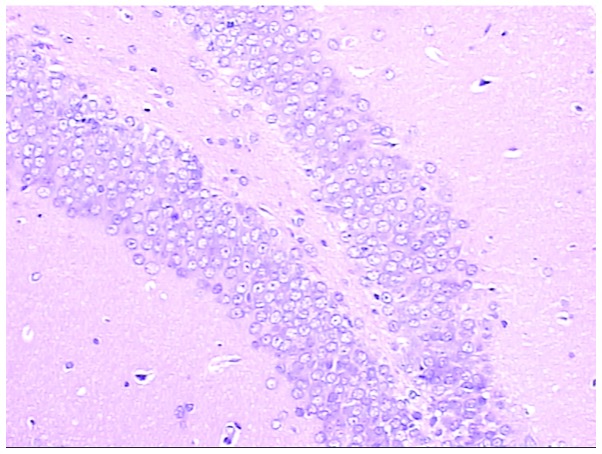
Hematoxylin and eosin staining showing the hippocampus of a rat at low altitude (magnification, ×100).

**Figure 5:**
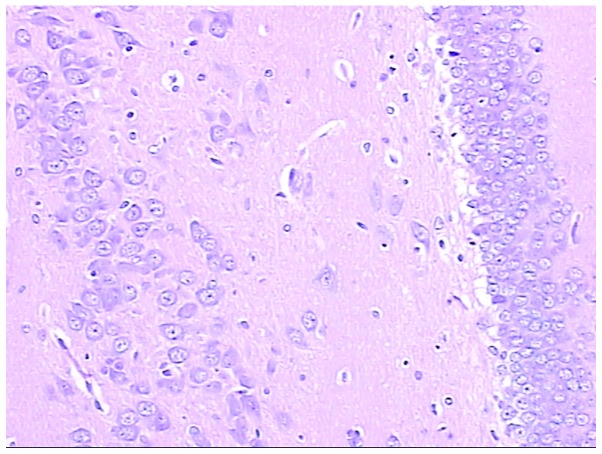
Hematoxylin and eosin staining showing brain neurons of a rat at low altitude (magnification, ×100).

**Figure 6:**
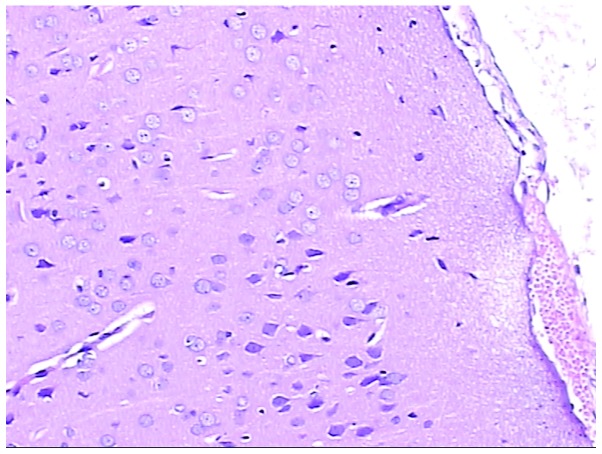
Hematoxylin and eosin staining showing brain neurons of a rat at high altitude (magnification, ×100).

**Figure 7:**
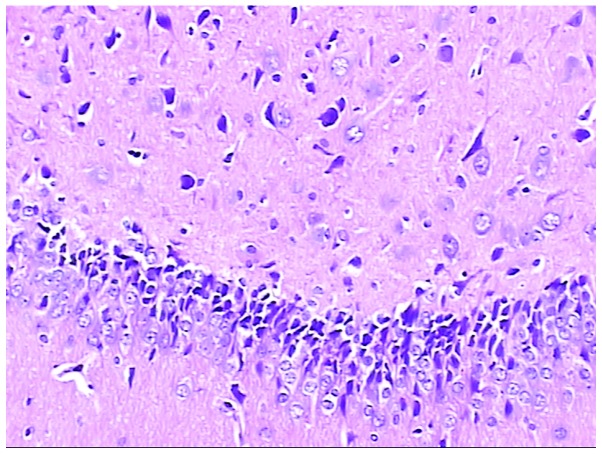
Ematoxylin-eosin staining showing the hippocampus of a rat at high altitude (magnification, ×100).

**Figure 8:**
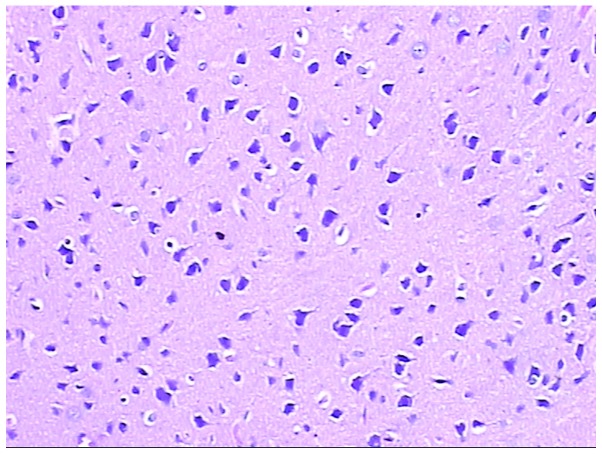
Hematoxylin and eosin staining showing brain neurons of a rat that returned to low altitude from high altitude (magnification, ×100).

**Figure 9:**
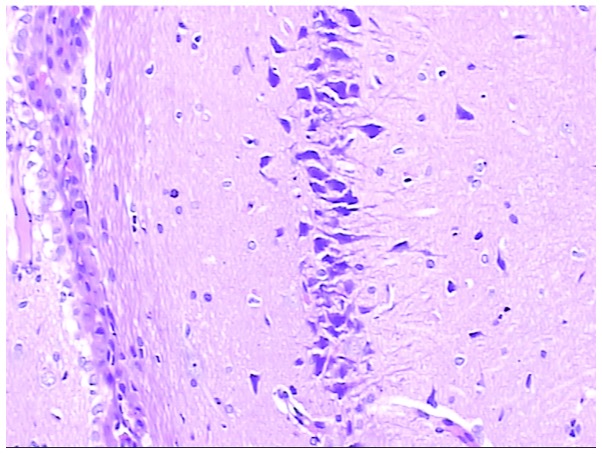
Hematoxylin and eosin staining showing the hippocampus of a rat that returned to low altitude from high altitude (magnification, ×100).

Figs. 4 and 5 show normal brain neurons and hippocampal tissue. Figs. 6 and 7 show pathological brain neurons and hippocampal tissue, respectively, following acute exposure to high altitude. It may be observed that the brain neurons were edematous and enlarged perivascular space was present, and the hippocampal neurons were metamorphic and karyopyknotic. Furthermore, Figs. 8 and 9 are pathological brain neurons and hippocampal tissues from rats that had returned to low altitude from high altitude, where brain neurons were edematous, metamorphic and karyopyknotic, and hippocampal neurons were metamorphic and karyopyknotic.

#### Results of hematoxylin and eosin staining of kidney tissue

**Figure 10:**
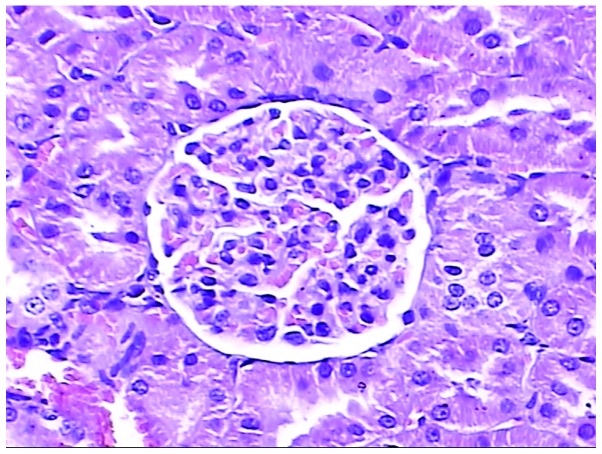
Hematoxylin and eosin staining showing normal kidney glomerulus tissue of a rat at low altitude (magnification, ×100).

**Figure 11:**
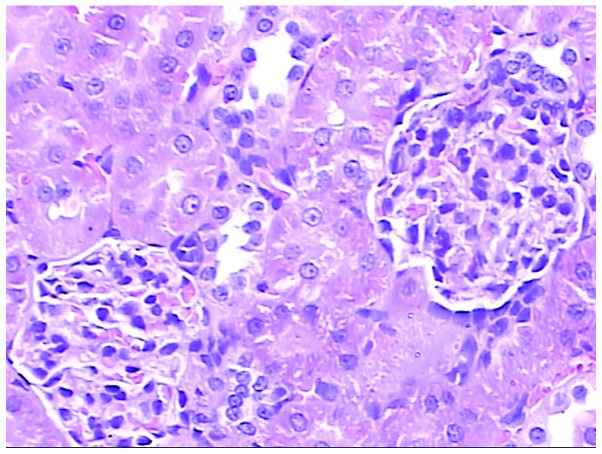
Hematoxylin and eosin staining showing normal kidney glomerulus tissue of a rat at high altitude (magnification, ×100).

**Figure 12:**
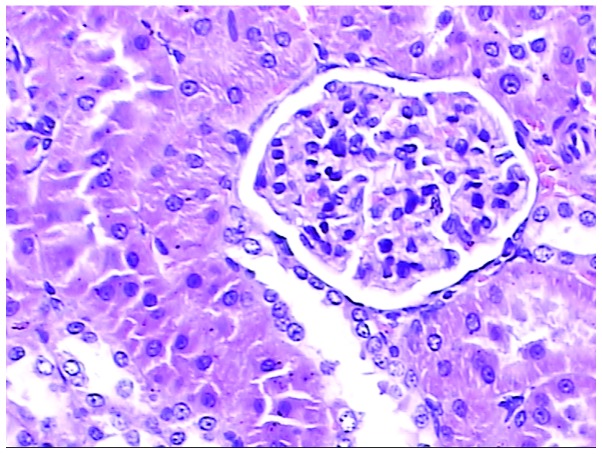
Hematoxylin and eosin staining showing normal kidney glomerulus tissue of a rat that returned to low altitude from high altitude (magnification, ×100).

In Fig. 10, normal kidney glomerulus tissue is shown in which the capillary nodes and glomera are clear with regular capsular spaces. Fig. 11 shows pathological kidney tissue following acute exposure to high altitude, in which hyperplastic mesangial cells can be observed. Fig. 12 shows pathological kidney tissues from rats that had returned to low altitude from high altitude. There were no evident changes, with the exception that that the mesangial cells were hyperplastic.

## Discussion

Blood gas analysis indicates that the blood pH was significantly decreased when the rats were acutely exposed to high altitude and also when the rats returned to low altitude from high altitude. When the blood pH is low, acidic drugs dissociate less (8) and the levels of molecular drugs, which have greater liposolubility and pass from the plasma into cells more easily, are increased. As a result, the distribution of acid drugs is likely to increase following acute exposure to high altitude and also when returning to low altitude from high altitude. Alkaline drugs are likely to be affected in a reverse manner. Therefore, it is necessary and of great value to investigate in greater detail the association between pH and the dissociation of drugs at high altitude.

A previous study (9) reported that Hb levels in rats at high altitude were higher than those in rats at low altitude. However, there was no significant difference in Hb levels between groups A and B in the present study. This may be attributable to the fact that the time span after acute exposure to high altitude was not long enough and it takes time for EPO levels to increase. In rats that were acutely exposed to high altitude and then returned to low altitude, the Hb level was lower than that in rats maintained at low altitude or at high altitude, and thus the capacity to carry oxygen was reduced. However, the pO_2_ and sO_2_ levels were similar to those in rats acutely exposed to high altitude. This may due to deadaptation (10) when rats return to low altitude from high altitude, or other reasons, which are worthy of further research in the future.

Blood gas analysis also indicated that the concentration of Na^+^ decreased at high altitude, which suggests that Na^+^ flowed into cells abundantly under anoxic conditions and this is likely to lead to cellular edema and capillary occlusion. Hypoxic microcirculation is thus exacerbated and would greatly affect drug disposition.

In the study by Gao *et al* (11), the K^+^ concentration in rats at high altitude was increased compared with that in rats at low altitude. K^+^ outflow causes the absence of intracellular K^+^, which is indispensable for protein synthesis and metabolism (including enzyme activity), which seriously affects the metabolism and excretion of drugs. In the present study, there was no significant difference in K^+^ concentration between the three groups. As for Cl^−^, in rats that were exposed to high altitude and then returned to low altitude, the Cl^−^ concentration increased compared with that of rats maintained at low altitude. Serum chloride plays a part in the synthesis of gastric acid (gastric acid levels increases following food intake, and serum chloride levels decrease) (12). In addition, chloride also takes part in renin secretion and adjustment (a reduction in serum chloride in the macula densa of the juxtaglomerular apparatus leads to inhibition of renin secretion, and verse versa). Serum chloride levels increase with dysbolism of sodium and acid base imbalance, which is in line with the present study. The results of the present study suggest that changes in the concentration of Cl^−^ are likely to affect digestion and absorption by the intestines and the functioning of kidneys, and further affect the absorption and excretion of drugs.

The results of the pathological examinations revealed that at high altitude, the alveolar walls were hyperemic, edematous and incrassate while the alveolar epithelium was hyperplastic with infiltrative neutrophilic granulocytes. The alveolar septa were widened. This suggests that oxygen exchange in the lungs becomes difficult, which is consistent with the blood gas analysis results. These pathological changes did not recover after the rats returned to low altitude from high altitude, which explains why there is no significant difference in the results of blood gas analysis, with the exception of K^+^ levels.

At high altitude, the concentration of serum Na^+^ was significantly decreased, which suggests that Na^+^ flowed into cells abundantly under anoxic conditions and led to cellular edema, capillary occlusion, and hypoxia of the microcirculation. As a result, pathological examination revealed pulmonary edema and cerebral edema, which is consistent with the blood gas analysis results. In conclusion, the blood gas index and pathological features of the rats were changed significantly at high altitude, which is likely to seriously affect drug absorption, distribution, metabolism and excretion and ultimately the therapeutic effects and adverse reactions of drugs.

Evident changes in the levels of LDH, ALP, TBIL and Cl^−^ were observed in the present study. The study conducted by Mei *et al* (13) found that LDH levels were markedly decreased in rats at high altitude. The study by Dai *et al* (14) showed that urea, creatinine, Cl^−^ and ALP levels were clearly decreased following acute exposure to high altitude, which is consistent with the present study, which found that LHD and TP levels were decreased by 58.44% and 26.82%, respectively, while TBIL and ALP were increased by 338.00% and 24.94%, respectively. These results suggest that the heart functions and hepatic functions of the rats changed following acute exposure to high altitude. Heart function has a great influence on blood rheology, blood pressure and circulation, thus affecting drug absorption and distribution. As the liver is the major organ responsible for drug metabolism, changes of liver function seriously affect the formation of metabolizing enzymes and their activities (15) thus altering drug distribution and metabolism in a hypoxic environment.

The reduction of total protein levels in the blood is likely to affect the protein binding rates of drugs, leading to increased levels of free drug in the blood and as a result, the drug concentration may be higher than the normal level in a hypoxic environment. Total protein levels rebounded when the rats were returned to low altitude, indicating that blood drug concentrations may be reduced when returning from high to low altitude. TBIL levels continued to rise while ALP and UA levels decreased upon return to low altitude, which suggests that liver function recovered to a certain extent, and its influence on drug metabolism was reduced when the rats returned to low altitude from high altitude. The study by Li (15) found that UA levels increased at high altitude. However, in the present study, the increase in UA levels at high altitude was not significant, whereas an increase of urea levels was evident when the rats returned to low altitude from high altitude, suggesting that the kidney function did not change much under acute hypoxia; however, changes were evident when the rats returned to low altitude from high altitude, which may be attributable to deadaptation (10,16). In the pathological observation, only minor changes in the kidneys were identified. However, since kidneys are the main path of drug excretion, although the results indicate that there was little injury of the kidneys in a hypoxic environment, drug excretion may be seriously affected in such an environment.

In conclusion, the kidneys are the main path of drug excretion, and have a great influence on the absorption, distribution, metabolism and excretion of drugs. There are changes in the elimination rate constant (Ke), peak time (Tmax), peak concentration (Cmax), half life (t_1/2_), clearance (CL) and area under the curve (AUC) associated with the drug metabolism and excretion and are thought to have a significant effect of the therapeutic and side effects of drugs (17). The degrees of influence require study in future experiments. Changes in pharmacokinetics have a close association with the therapeutic effects and adverse reactions of drugs, making it necessary to adjust the dose and usage of commonly used drugs at high altitude. This study may provide a basis and new ideas for clinical pharmacy at high altitude, for improving clinical medication and avoiding adverse reactions, in order to achieve personalized medication at high altitude.

## Figures and Tables

**Table I:** Comparison of blood gas analysis (mean ± standard deviation; n=7).

Blood gas indicators	Group A	Group B	Group C
pH	7.40±0.03^b^,^c^	7.22±0.17^a^	7.16±0.07^a^
BB (mmol/l)	−0.84±0.91^b^,^c^	−6.15±3.89^a^	−5.40±2.48^a^
BE (mmol/l)	−1.11±0.90^b^,^c^	−4.57±3.49^a^	−4.4±1.96^a^
ctCO_2_ (mmol/l)	24.74±0.80^b^	21.90±1.30^a^,^c^	23.5±0.68^b^
sO_2_ (%)	92.5±0.97^b^,^c^	7.97±4.68^a^	6.57±5.52^a^
pCO_2_ (mmHg)	38.32±3.56^b^,^c^	56.5±20.24^a^	67.25±10.34^a^
pO_2_ (mmHg)	83.04±2.88^b^,^c^	19.75±15.94^a^	8.75±6.13^a^
Hb (g/dl)	12.22±0.37^c^	11.15±1.99^c^	8.27±1.02^a^,^b^
cNa^+^ (mmol/l)	144.57±0.78^b^,^c^	140.50±4.50^a^	138.5±2.51^a^
cK^+^ (mmol/l)	5.07±0.34	4.97±0.41	5.27±0.73
cCl^−^ (mmol/l)	99.28±0.75^b^,^c^	106.00±3.91^a^	103.50±2.38^a^

BB, buffer base; BE, base excess; ctCO_2_, content of total carbon dioxide; sO_2_, oxygen saturation of arterial blood; pCO_2_, carbon dioxide tension of arterial blood; pO2, oxygen tension of arterial blood; Hb, hemoglobin; cNa^+^, concentration of sodium ions; cK^+^, concentration of potassium ions; cCl^−^, concentration of chloride ions.

a P<0.05 compared with group A,

b P<0.05 compared with group B and

c P<0.05 compared with group C.

**Table II:** Comparison of biochemical indicators (mean ± standard deviation; n=7).

Biochemical indicators	Group A	Group B	Group C
LHD (U/l)	873.5±186.13^b^,^c^	363.00±116.25^a^	297.20±99.64^a^
AST (U/l)	138.14±13.43	163.00±8.18	160.80±30.76
ALT (U/l)	54.71±5.9^c^	65.66±14.36	72.00±5.24^a^
TP (g/l)	64.85±2.67^b^,^c^	47.46±6.59^a^,^c^	53.56±9.22^a^,^b^
TBIL (μmol/l)	0.65±0.26^b^,^c^	2.85±0.45^a^,^c^	3.76±0.37^a^,^b^
ALP (U/l)	240.28±22.23^b^	300.20±34.81^a^,^c^	242.60±23.75^b^
GLU (mmol/l)	5.11±1.05	6.20±2.99	6.80±0.96
Urea (mmol/l)	5.11±1.05^c^	4.96±1.03^c^	6.96±1.46^a^,^b^
UA (μmol/l)	77.14±7.56	84.82±30.36	74.12±17.12

LHD, lactate dehydrogenase; AST, aspartate aminotransferase; ALT, alanine aminotransferase; TP, total protein; TBIL, total bilirubin; ALP, alkaline phosphatase; GLU, glucose; UA, uric acid.

a P<0.05 compared with group A,

b P<0.05 compared with group B and

c P<0.05 compared with group C.
